# New Insights into Cerebrovascular Pathophysiology and Hypertension

**DOI:** 10.1161/STROKEAHA.121.035850

**Published:** 2022-03-08

**Authors:** Alastair JS Webb, David J Werring

**Affiliations:** * Wolfson Centre for Prevention of Stroke and Dementia, University of Oxford, UK; ┼ Stroke Research Centre, UCL Queen Square Institute of Neurology, London, UK

## Abstract

Despite advances in acute management and prevention of cerebrovascular disease, stroke and vascular cognitive impairment together remain the world’s leading cause of death and neurological disability. Hypertension and its consequences are associated with over 50% of ischaemic and 70% of haemorrhagic strokes but despite good control of blood pressure there remains a 10% risk of recurrent cerebrovascular events and there is no proven strategy to prevent vascular cognitive impairment.

Hypertension evolves over the life-span, from predominant sympathetically-driven hypertension with elevated mean blood pressure (BP) in early and mid-life to a late-life phenotype of increasing systolic and falling diastolic pressures, associated with increased arterial stiffness and aortic pulsatility. This pattern may partially explain both the increasing incidence of stroke in younger adults as well as late-onset, chronic cerebrovascular injury associated with concurrent systolic hypertension and historic midlife diastolic hypertension. With increasing arterial stiffness and autonomic dysfunction, blood pressure variability increases, independently predicting the risk of ischaemic and intracerebral haemorrhage, and is potentially modifiable beyond control of mean blood pressure.

However, the interaction between hypertension and control of cerebral blood flow remains poorly understood. Cerebral small vessel disease is associated with increased pulsatility in large cerebral vessels and reduced reactivity to carbon dioxide, both of which are being targeted in early-phase clinical trials. Cerebral arterial pulsatility is mainly dependent upon increased transmission of aortic pulsatility via stiff vessels to the brain whilst cerebrovascular reactivity reflects endothelial dysfunction. In contrast, although cerebral autoregulation is critical to adapt cerebral tone to blood pressure fluctuations to maintain cerebral blood flow, its role as a modifiable risk factor for cerebrovascular disease is uncertain.

New insights into hypertension-associated cerebrovascular pathophysiology may provide key targets to prevent chronic cerebrovascular disease, acute events and vascular cognitive impairment.

## Introduction

Hypertension is the risk factor with the highest global attributable risk for death and the second-highest attributable risk for morbidity.^1^ It affected over a quarter of the world’s population in 2000 and is set to rise to approximately 29% of the population by 2025, affecting 1.56 billion people.^2,3^ A mean systolic blood pressure (SBP) >115mmHg explains 50-60% of the population attributable risk of ischaemic stroke^4,5^ and over 70% of haemorrhagic stroke,^6^ whilst an increment of 20mmHg SBP doubles the risk of stroke between 40-69 years of age.^4^ Randomisation to a treatment which reduces mean SBP results in a significant reduction in vascular events with a 5-6mmHg decrease in diastolic pressure resulting in a 33-50% reduction in stroke and a 4-22% reduction in acute coronary events.^7^

However, a detailed understanding of the mechanistic relationship between hypertension and stroke has lagged behind that of other vascular outcomes due to heterogeneity in stroke aetiology,^8^ heterogeneous radiological manifestations of chronic cerebrovascular injury^9^ and a lack of stroke phenotyping in observational cohort studies and blood-pressure lowering clinical trials.^10^ There have been limited studies of the effects of hypertension on the cerebral circulation^11^ and there are no specific proven hypertension-related treatments for small vessel disease (SVD), beyond blood pressure lowering.

Hypertension is associated with all ischaemic stroke subtypes, whether cardioembolic stroke related to atrial fibrillation (AF),^12^ large artery stroke due to carotid stenosis,^13^ lacunar stroke in SVD^14^ or less common stroke aetiologies such as carotid dissection,^15^ with an even more powerful effect for intracerebral haemorrhage. These aetiologies depend upon intermediate disease phenotypes which can be treated, such as carotid endarterectomy for atherosclerosis^16^ or anticoagulation for AF.^17^ However, the intracranial physiological effects of hypertension are less well understood even though they may explain some of the residual risk of recurrent stroke despite effective BP lowering. In the PROGRESS study, a 12mmHg reduction in blood pressure with combination treatment reduced recurrent stroke by up to 43%,^18^ whilst any treatment reduced intracerebral haemorrhage by 50%, with similar efficacy in all secondary prevention trials,^19^ but there was still a 10% risk of recurrent events. In the multi-centre TIARegistry, there was a 14.3% risk of recurrent major vascular events (MACE) at 5 years with a 9.5% risk of recurrent stroke,^20^ despite high-quality secondary prevention.

**Figure 1:**
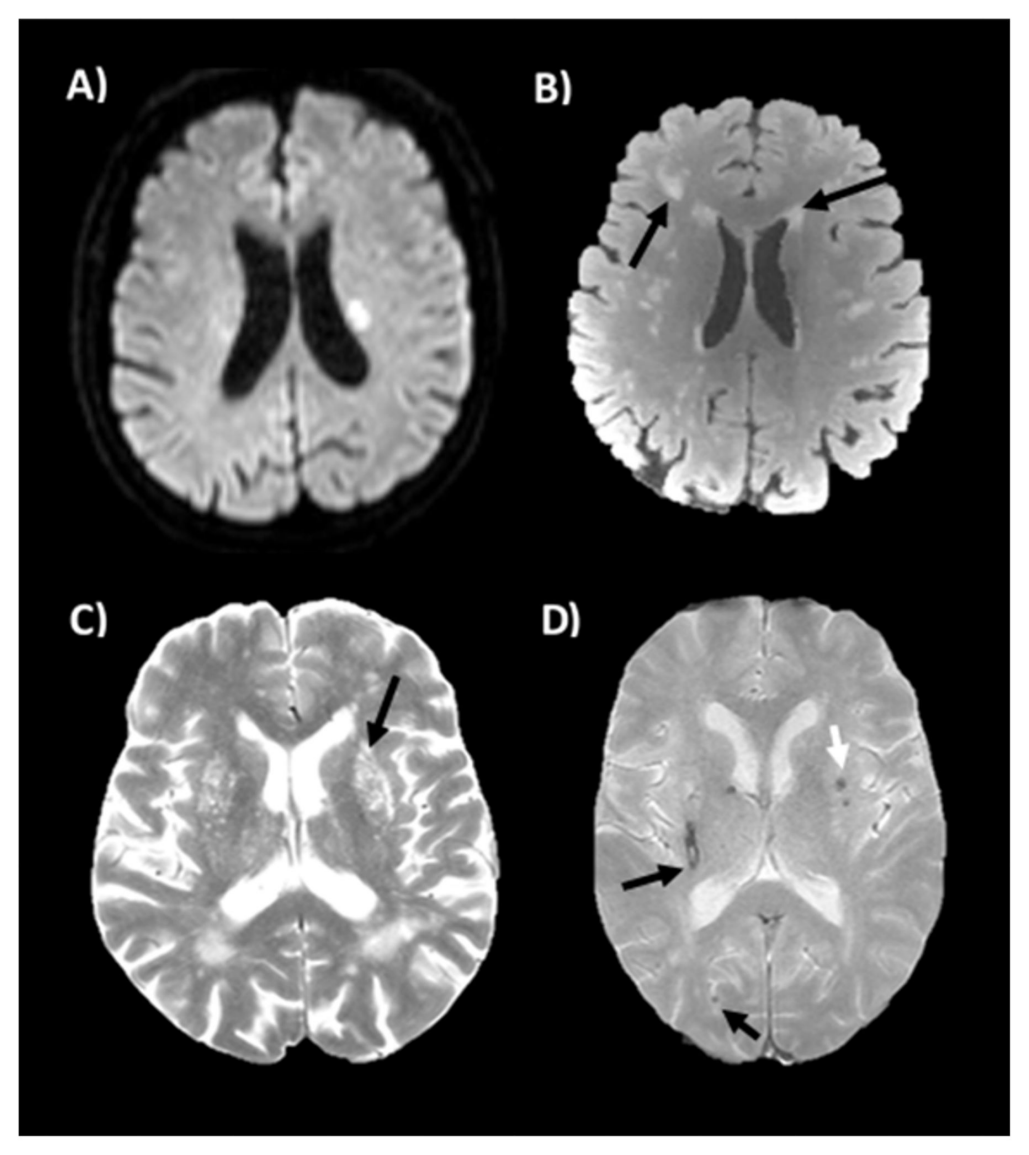
Radiological manifestations of small vessel disease. A) An acute small subcortical infarct on DWI imaging; B) Deep and periventricular white matter hyperintensities (arrowed); C) Dilated perivascular spaces bilaterally in the basal ganglia (arrowed on the left); D) a slit-like haemosiderin-lined cavity due to a chronic right intracerebral haemorrhage, subcortical (deep) cerebral microbleeds (white arrow) and a cortical (lobar) (black arrow) microbleed.

The principal manifestation of chronic cerebrovascular injury is cerebral small vessel disease. Originally defined by pathological changes including vessel lipohyalinosis, fibrinoid necrosis and vessel rarefaction, cSVD is now primarily defined by imaging characteristics on MRI (figure 1) for both of its commonest two forms: cerebral amyloid angiopathy (CAA) and deep perforator arteriopathy.^9^ These represent further intermediate phenotypes linking hypertension with cerebrovascular outcomes, and are responsible for 30% of ischaemic strokes, 80% of intracerebral haemorrhage^21,22^ and 45% of dementia, as well as late-onset refractory depression and significant global functional impairment at older ages.^23,24^ SVD is evident on brain imaging in >50% of people over 65 years of age and nearly all people over 80 years of age^25^. However, no specific treatments exist to target its progression.

Any original data that support the conclusions of this study are available from the authors upon reasonable request.

### Epidemiological insights

Derivation of the statistical measure ‘usual’ blood pressure in the early 1990s allowed precise estimation of the blood pressure related vascular risk by adjusting for variation between clinic visits, to remove the resulting bias that caused underestimation of the effect of average blood pressure (‘regression dilution bias’).^7,26^ Randomised controlled trials then confirmed that a reduction in mean blood pressure reduced the risk of recurrent events by the predicted degree,^7^ irrespective of baseline cerebrovascular risk^18^ and at minimally increased baseline blood pressure levels.^27,28^ However, hypertension research for primary prevention has lacked a nuanced focus on stroke, with limited stroke phenotyping in large randomized controlled trials (RCTs), whilst stroke research has lacked a nuanced understanding of hypertensive physiology and its effect on the cerebral circulation.

#### Blood pressure evolution over the lifespan

At all ages, elevated mean blood pressure is associated with an increased risk of stroke.^10^ As preventative strategies including antihypertensive treatment and reduced salt intake have become more effective the incidence of stroke has reduced in older age groups.^29^ However, due to the aging population there is a marked overall increase in the prevalence of stroke and vascular cognitive impairment. Furthermore, in younger patients under 50 years, particularly women, age-standardised stroke incidence is increasing, associated with increasing rates of obesity and hypertension.^30,31^

**Figure 2:**
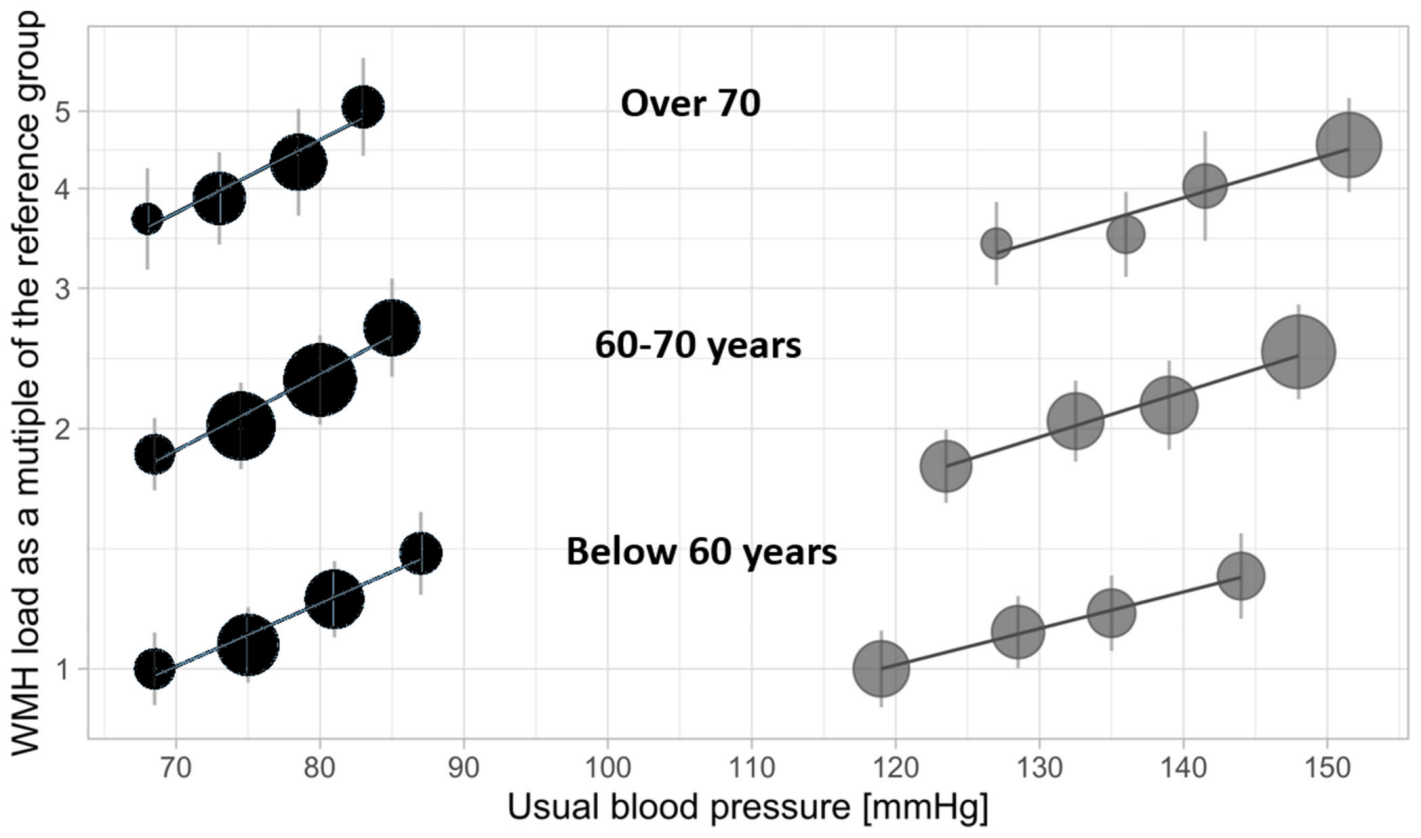
Relationship between ‘Usual’ diastolic and systolic blood pressure and white matter hyperintensities in the UK Biobank Study, stratified by age group. Severity of white matter hyperintensities is expressed as the ratio of their volume to the average volume in the youngest patients with the lowest blood pressure, plotted on a logarithmic scale. *Wartolowska K, Webb AJS. EHJ (2020)*.

In late life, stroke, SVD and vascular cognitive impairment reflect the cumulative cerebrovascular effect of hypertension over the whole life-span (figure 2). Although SBP rises linearly with age, at approximately 55 years of age diastolic blood pressure starts to fall, with a shift from mixed or diastolic hypertension to an increased frequency of isolated systolic hypertension. This reflects increasing aortic stiffness and the cerebrovascular effects of long-standing hypertension.^32^

Similarly, SVD is uncommon before 55 years of age but then increases at an increasing rate,^25^ driven by aging and hypertension and with a strong, genetic contribution.^33^ White matter hyperintensities (WMH) and concurrent systolic blood pressure are strongly associated at all ages,^34^ but there is a stronger relationship with midlife blood pressures,^35,36^ whilst the strength of the relationship increases the earlier in life the blood pressure is measured.^37^ Early microstructural brain imaging changes on diffusion tensor imaging in midlife participants in the UK Biobank study are cross-sectionally associated predominantly with mean arterial pressure, independent of age, whilst the association with pulse pressure attenuates significantly after adjustment for age. However, this interaction is synergistic such that increased pulse pressure accentuates the age-related deterioration in cerebral microstructure.^36^

In contrast to the long-term association with chronic SVD, recent blood pressure elevations are more strongly associated with both acute intracerebral haemorrhage^38^ and acute ischaemic stroke due to small vessel occlusion,^39^ implying a potentially causative interaction between increasing blood pressure levels over a timescale of a year or less, and both rupture or occlusion of small cerebral vessels. This relationship is particularly strong for stroke subtypes most closely related to hypertension, including deep parenchymal vs lobar haemorrhage and lacunar versus non-lacunar stroke, and provides a strong rationale for early, consistent blood pressure control, particularly after intracerebral haemorrhage, as is being tested with telemetric home monitoring in PROHIBIT-ICH (NCT03863665).

Mendelian randomisation has also demonstrated an age-dependent evolution of the relationship between blood pressure and cerebrovascular outcomes, with a likely causative association between high blood pressure and all stroke subtypes, except for lobar intracerebral haemorrhage.^40^ However, there were age-dependent differences between genetically determined drivers of mean versus pulsatile blood pressure. Both were associated with late-life large artery athero-thrombo-embolism, but elevated mean pressure was dominant across ages for other aetiological subtypes.^41^ Similar analyses have confirmed likely causative relationships between hypertension and the risk of incident all-cause dementia.^42^

#### Early life determinants of hypertension and cerebrovascular pathology

Early-life risk factors for late life cerebrovascular disease include low birth weight and pre-eclampsia,^43^ but vascular risk factors remain dominant after childhood.^44^ In particular, overactivity of the sympathetic nervous system is a key driver of hypertension, with increased muscle sympathetic nerve activity (MSNA) associated with obesity and blood pressure.^45^ This implies a potential treatment target with early, unblinded trials of renal sympathetic denervation (RDN) suggesting large reductions in systolic blood pressure (up to 40mmHg). Unfortunately, the properly blinded, sham-controlled Symplicity HTN-3^46^ study failed to replicate this. However, more recent trials (SPYRAL-ON and OFF-MED, RADIANCE-HTN),^47^ with newer devices and 4-quadrant denervation, have suggested that a more rigorous interventional approach can achieve significant blood pressure reductions. However, the achieved BP reductions are equivalent only to the addition of one new drug, whilst the application of RDN to patients with stroke is yet to be tested.

Baroreceptor activation therapy is an alternative, whereby an implanted stimulator increases the parasympathetic:sympathetic balance, with a resulting reduction in blood pressure. Lagging behind RDN, uncontrolled studies have been prone to bias but a sustained blood pressure reduction was achieved in the sole RCT in resistant hypertension (RHEOS PIVOTAL).^48^

#### Arterial stiffness, pulse wave veloctity and wave reflection

Late-life hypertensive phenotypes are characterized by increasing arterial stiffness, with a reduction in elastin and increase in collagen in the aorta reducing vascular compliance and increasing speed of travel of the aortic pressure wave from the heart to the carotid and femoral arteries (pulse wave velocity, PWV). This marker of ‘vascular aging’ has a hazard ratio of 1.54 per standard deviation increase for stroke and 1.42 for all vascular events,^49–51^ and its progression is hypertension-dependent, with a 20mmHg elevation of SBP associated with a 1.14m/s progression in PWV per decade.^52^ Furthermore, there are strong associations between aortic PWV and specific cerebrovascular phenotypes including acute lacunar stroke,^53^ deep intracerebral haemorrhage (by contrast with lobar),^54^ cerebral SVD and cognitive impairment ^55,56^, with a similar odds ratio of 1.25-1.3 per SD increase in PWV associated with an increase in severity of white matter hyperintensities on the Fazekas scale.

Increased arterial stiffness is also associated with increased wave reflection, whereby the pressure-wave generated with each cardiac impulse ‘reflects’ from points of increased impedance, either at the junction of large and medium vessels or between medium arteries and arterioles.^57^ Following reflection, the ‘reverse’ travelling wave then returns towards the heart to meet the forward travelling wave before it has dissipated. With increasing arterial stiffness, there is thus greater ‘augmentation’ of the peak of the waveform, increasing the aortic systolic pressure. Aortic BP is even more strongly associated with the risk of recurrent events than brachial pressure,^58^ since low resistance vascular beds (renal and cerebral) are more directly exposed to aortic pressures. Thus, a greater reduction in aortic pressure with an amlodipine-based regimen compared to an atenolol-based regimen potentially explained differences in vascular outcomes in the ASCOT-BPLA trial.^59^

#### Blood pressure variability

Mean blood pressure level over the lifespan is the strongest predictor of cerebrovascular disease.^4^ However, the statistical adjustment used to calculate ‘usual’ blood pressure depends upon adjusting for the variation in BP between assessments. The greater the variability in blood pressure (BPV) the greater the adjustment and the stronger the estimated relationship between mean blood pressure and subsequent risk. However, blood pressure is highly variable between clinic visits,^60^ and across five cohorts, the top decile of visit-to-visit BP variability was associated with 2.2-3.8 times the risk of recurrent stroke compared to the bottom decile.^61^ Variability was reduced in patients randomized to a calcium-channel blocker based regimen compared to an atenolol-based regimen, explaining the difference between treatment regimens that was not explained by mean blood pressure^62^ and consistent with effects of calcium channel blockers or thiazide-like diuretics in all large randomized controlled trials BPV and the risk of stroke.^63^

**Figure 3:**
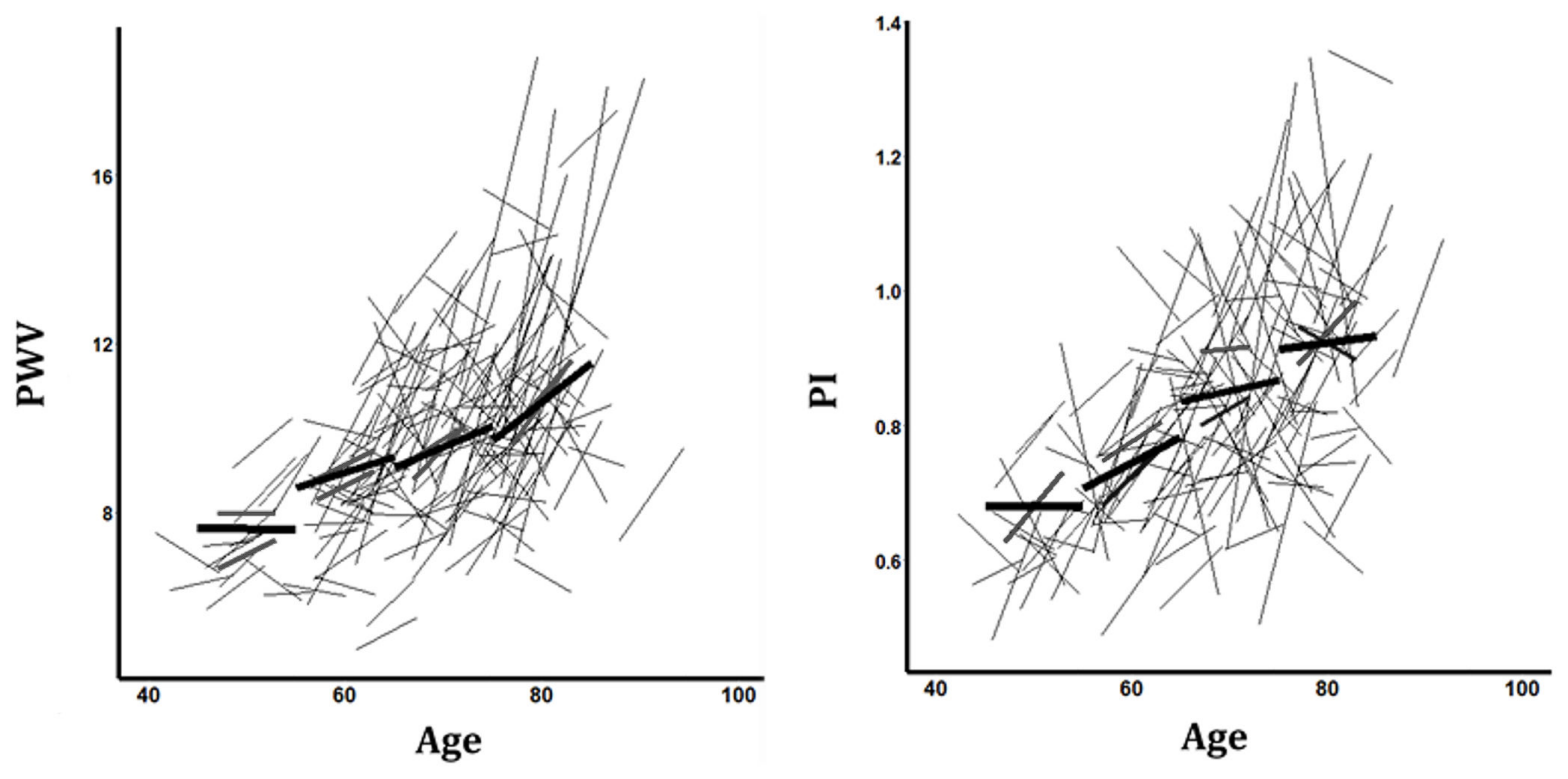
Rate of progression of aortic pulse wave velocity (PWV) and middle cerebral pulsatility (PI) over 5 years in 188 patients after TIA or minor stroke, stratified by quartiles of age. Individual changes are show, with summary results derived from a mixed effect linear model, stratified by quartiles of age and subdivided into men (red) and women (blue).^56^

Visit-to-visit BP variability has subsequently been found to predict conditions from renal failure to dementia, with consistent associations with vascular risk^64^ on both visit-to-visit and home day-to-day variability,^65^ and worse outcome after both ischaemic^66^ and haemorrhagic stroke^67^ with increasing BPV. The association between BPV and cognitive impairment is greater than that for mean BP, although associations are highly heterogeneous between studies and may reflect reverse causation.^68^ Variability is predictive from visit-to-visit, day-to-day^65,69^ or beat-to-beat,^70^ is associated with arterial stiffness, baroreceptor dysfunction and increased reactivity to stress^71^ and progresses over the age of 55^72^ in parallel with progression of arterial stiffness and aortic pulsatility (figure 3).

The association between BPV, increased SVD and cognitive impairment^73^ supports the importance of an understudied, late-life complex phenotype of severe cerebral SVD, cognitive impairment, increased arterial stiffening and greater blood pressure variability. In this group, there may also be under-appreciated vulnerability to hypoperfusion of the brain due to low blood pressures,^74^ as suggested by the U-shaped association between cognitive impairment and diastolic blood pressure and the relationship with postural hypotension.^75^ In a recent analysis from the ARIC study, the highest risk of dementia was seen in patients with high mid-life blood pressure but late life hypotension.^76^

### Pathophysiology of hypertensive cerebrovascular disease

**Figure 4:**
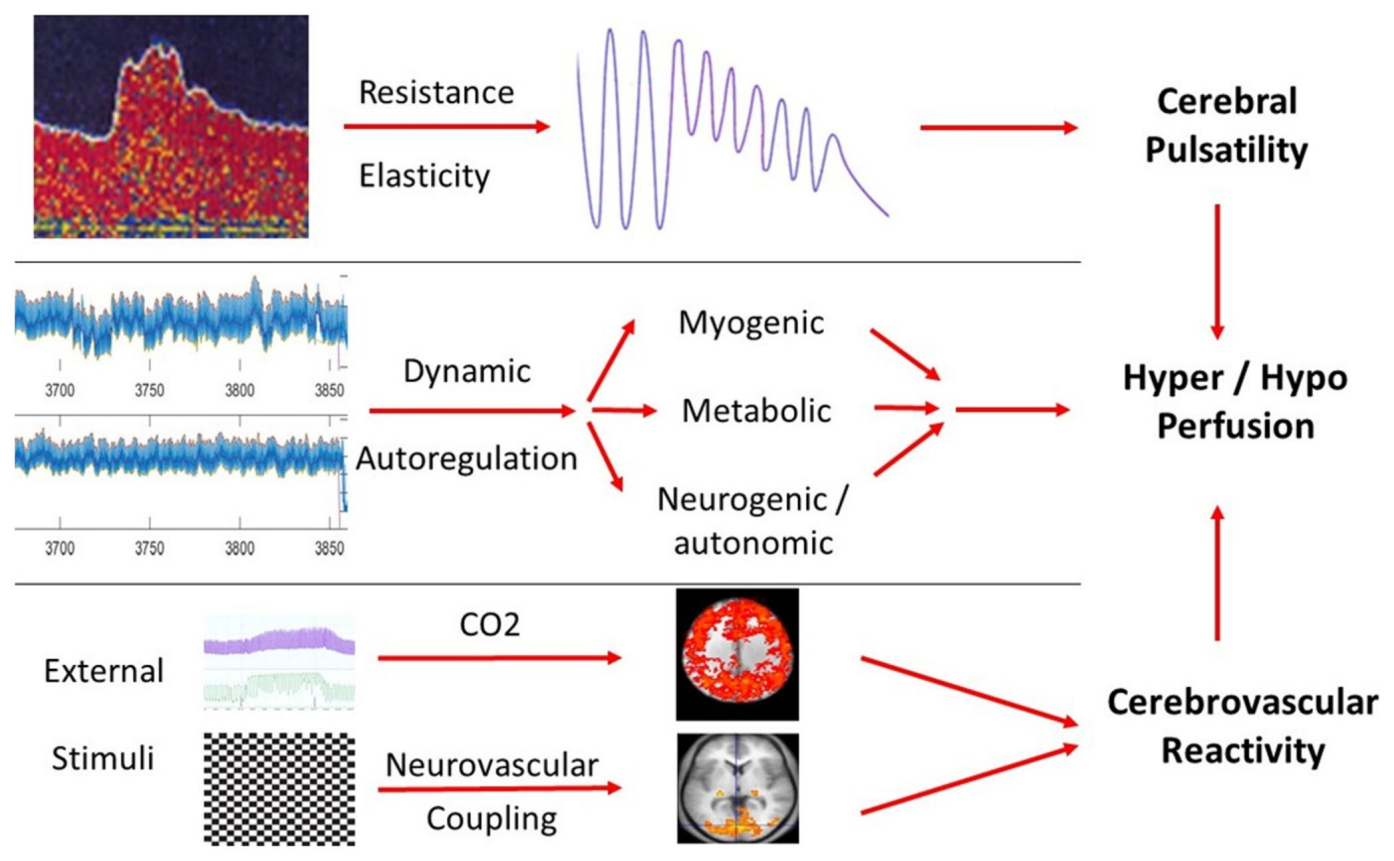
Major mechanisms of cerebrovascular adaptation to changes in blood pressure and demand for blood flow to control cerebral perfusion.

Hypertension has broad vascular effects. It increases cardiac load, coronary artery disease and atrial dilatation, leading to AF, ventricular wall dyskinesia and heart failure. The increased shear stress on the endothelium contributes to the induction of atherosclerosis and carotid stenosis. These disease phenotypic manifestations have led to specific treatments to reduce stroke risk in the context of AF or carotid atherosclerosis. However, the intracranial effects of hypertension, particularly on cerebral small vessels, have not yet resulted in equivalent intermediate treatment targets, despite multiple identified pathways modifying the effect of hypertension and control of blood flow to the brain (figure 4).

#### Pathophysiological changes of hypertension

Chronic hypertension is associated with characteristic changes in small vessels throughout the body. Shear stress promotes intracranial atherosclerosis and intracranial stenoses, whilst in smaller vessels it induces smooth muscle hypertrophy, reorganization (or disorganization) of smooth muscle cells, a reduction in the wall:lumen ratio, reduced vascular compliance and, over time, rarefaction of vessels. This increases intracranial cerebrovascular resistance, balancing increased perfusion pressures, but is associated with impaired resting perfusion is evident in the white matter of patients with severe cSVD.^74^ This hypertrophic remodeling is partly dependent upon sympathetic nerve fibre innervation, and ameliorated (in rat models) by antagonism of the angiotensin-aldosterone system. Other potential targets include PPARy receptors, with reduced remodeling of large cerebral vessels in rat models under the influence of rosiglitazone or pioglitazone,^77^ with could explain the reduction in cerebrovascular events with pioglitazone in the IRIS trial.^78^

Endothelium derived nitric oxide (NO) is a key driver of vasodilatation, with reduced functional hyperaemia in older hypertensive patients, reduced vascular reactivity in cerebral small vessel disease and increased circulating markers of endothelial cell dysfunction in patients with cSVD and hypertension.^79^ Furthermore, break down of the blood brain barrier (BBB) in cerebral small vessel disease is seen both in white matter hyperintensities and in areas of normal appearing white matter in patients with either lacunar stroke or established white matter hyperintensities elsewhere in the brain.^80^ BBB dysfunction may be induced directly by chronic hypertension as a potential mechanism for development of SVD or cognitive decline, either through haemodynamic effects or through the induction of oxidative stress and low-grade inflammation^81^ or through Amyloid beta-mediated pathways.^82^

#### Phenotypes of Cerebral Small Vessel disease

Cerebral small vessel disease is associated with 30% of ischaemic stroke, 80% of intracerebral haemorrhage and 45% of dementia.^39^ Recurrent stroke was reduced in patients with lacunar events in the SPS3 trial, whilst haemorrhagic stroke was reduced by 63%. Moreover, intensive blood pressure lowering was associated with a reduction in progression of white matter hyperintensities (PROGRESS, SPRINT-MIND; ACCORD-MIND), with a dose-response relationship between blood pressure reduction and rate of progression. However, there was also increased cerebral atrophy, and in the majority of these trials baseline white matter hyperintensities were not severe, limiting generalizability to patients with advanced cerebral SVD. However, reassuringly, there was no evidence of impairment of MRI-measured cerebral perfusion with intensive treatment in the more severely affected participants in the PRESERVE trial.^83^

Cerebral microbleeds are the most prevalent haemorrhagic radiological manifestation of cerebral SVD. These areas of ‘blooming’ artefact most often reflect deposition of paramagnetic material, usually microscopic areas of subclinical blood extravasation (haemorrhage), though a range of lesions from haemosiderin deposition to intact erythrocytes to vessel wall calcification have been demonstrated.^84,85^ The pattern of distribution of microbleeds is indicative of the underlying pathology, with a strictly lobar distribution associated with cerebral amyloid angiopathy, reflecting deposition of β-amyloid in small cortical and leptomeningeal vessels. In contrast, deep microbleeds in the basal ganglia and brainstem are associated with hypertension, left ventricular hypertrophy, arterial stiffening and pulse pressure with a dose-response relationship between hypertension and severity of microbleeds.^86^ Although hypertension may trigger symptomatic lobar haemorrhage in CAA, there is a weaker overall association with hypertension, suggesting hypertension may aggravate CAA but not cause it. Studies to intensively control blood pressure after haemorrhage, either by intensive monitoring (PROHIBIT-ICH) or early triple, combination therapy (TRIDENT), may help to determine the effects of improved hypertension control.

Similar to cerebral microbleeds, there is a stronger association between hypertension and dilated perivascular spaces in the basal ganglia than with PVS in the centrum semiovale.^87^ This distribution of SVD markers associated with hypertension reflects brain regions most exposed to intraarterial pressures and pulsatility,^88^ and vulnerability to these factors is at least partially genetically determined, with a heritability of basal ganglia PVS of up to 0.6 and significant shared heritability with white matter hyperintensities and shared genetic associations in large scale genome-wide association studies.^89^ This indicates a common hypertension-associated genetic predisposition across different manifestations of cSVD, and may also contribute to observed associations between PVS and both ischaemic^90^ and intracerebral haemorrhage.^91^

#### Cerebral Pulsatility

Cerebral vessels modify the blood pressure to maintain perfusion of the brain throughout the cardiac cycle (via arterial pulsatility), from beat-to-beat and hour-to-hour (via cerebral autoregulation) and in response to endogenous and exogenous stimuli (via reactivity). Cerebral artery pulsatility is measured on transcranial ultrasound (Gosling’s pulsatility index), 4d-phase contrast MRI sequences,^92^ or by high temporal-resolution multiband imaging.^93^ Cerebral pulsatility is strongly associated with severity of white matter hyperintensities,^94^ acute lacunar infarcts, cerebral microbleeds^95^ and dilated perivascular spaces.^96^ It increases with age and is tightly associated with aortic stiffness and aortic pulsatility,^56,97^ reflecting increased transmission of the aortic waveform to the brain, modified by dampening of the waveform at points of tortuosity and impedance and by the distal resistance of smaller cerebral vessels. It is highly reproducible, progresses over the age of 65 and independently predicts the risk of recurrent cerebrovascular events. However, methods to modify it independent of mean blood pressure are not clear. In the ECLIPSE study a phosphodiesterase 3 inhibitor (cilostazol)^98^ reduced cerebral pulsatility, potentially explaining beneficial effects of cilostazol in clinical trials beyond its known antiplatelet activity.^11^

#### Perivascular Clearance

Arterial pulsatility may also be key to drainage of interstitial fluid from the brain along perivascular spaces, sometimes called ‘glymphatics’. This recently identified pathway challenged the axiom that CSF reabsorption only happened via the arachnoid granulations, with intracisternal injection of radiolabeled tracers appearing in the CSF filled perivascular spaces around arteries, and then around veins,^99^ exiting via newly identified meningeal lymphatic vessels.^100^ This pathyway has been linked to drainage of amyloid β and other solutes. Despite limitations in experimental techniques^101^, 2-photon imaging tracer studies and ligation studies of the carotid^102^ indicate that arterial pulsatility provides motive force for perivascular drainage in healthy animals. However, it is unclear whether hypertension-associated increases in pulsatility and vascular stiffness affect perivascular drainage in humans, given a lack of in vivo glymphatic imaging techniques that do not require intrathecal injection of contrast.^103^

#### Cerebrovascular Reactivity

The endothelium controls the cerebrovascular response to stimuli, regulating smooth muscle tone via balanced vasodilator and vasoconstrictor pathways. The archetypal, eNOS vasodilator pathway induces smooth muscle cGMP upregulation and cell relaxation to increase local CBF, which antagonises vasoconstrictor pathways such as endothelin-mediated vasocontriction.

Cerebrovascular reactivity (CVR) is most commonly measured in vivo by altering carbon dioxide levels, (inhalation of CO2, breath-holding, hyperventilation, acetazolamide) and measuring the vascular response (BOLD-MRI, ASL-MRI, MCA cerebral blood flow velocity on TCD, near infrared spectroscopy).^104^ CVR is an attractive therapeutic target, being simple to measure, modifiable by established medications, and with a biologically plausible relationship to disease. It is associated with severity of white matter hyperintensities, dilated perivascular spaces^105^ and microbleeds, and progresses in parallel with progression of SVD. Recent studies have also demonstrated impaired CVR in the basal ganglia in patients with deep intracerebral haemorrhages.^106^

However, studies of the relationship between hypertension and CVR, independent of established cerebrovascular injury remain sparse with inconsistencies in methods of measurement of CVR, limited data in different cerebrovascular manifestations and uncertainty regarding the temporal order of the components of the phenotypes. This critical question may be answered by large collaborative studies such the MarkVCID and SVDs@TARGET consortia, and by interventional studies targeting improvements in CVR: TREAT-SVDs (NCT03082014), OxHARP (NCT03855332), LACI-2 (NCT03451591).

#### Cerebral Autoregulation

Cerebral autoregulation maintains cerebral blood flow despite changes in blood pressure, with sustained BP elevation inducing vasoconstriction and increased resistance to blood flow. Hypertension is associated with a rise in the ‘set-point’ of this curve to compensate for elevations in blood pressure, although the dynamic range achieved may still be less than the upper limit of blood pressure variability.

With technical advances to measure beat-to-beat blood pressure and cerebral blood flow, dynamic cerebral autoregulation (dCA) reflects rapid adaptation to changes in blood pressure.^107^ dCA is measured either by induced blood pressure changes (thigh-cuff release, pharmacologically-induced hypertension) or by resting-state relationships between spontaneous fluctuations in blood pressure and cerebral blood flow in time-domain (‘Mx’), or frequency-domain transfer function analysis to estimate the magnitude (gain), delay (phase) and strength of the relationship (coherence), or by modeling the response to an acute step change (autoregulation index, ARI). The underlying changes in vascular tone depend upon myogenic, metabolic and autonomic/neurogenic responses to maintain stable CBF.

Despite the critical biological importance of cerebral autoregulation, evidence for its role in the aetiology of stroke is limited. Autoregulation is commonly impaired in the ipsilateral hemisphere in severe ischaemic stroke,^108^ is associated with an increased risk of early neurological deterioration^109^ and haemorrhage^110^ and can predict a poor response to reperfusion therapies despite recanalisation,^111^ but studies have been small with inconsistent findings, partly due to difficulties in consistent measurement of dCA, although international collaborations (INFOMATAS) seek to better define its role. ^112^

In comparison, the population-based effect of hypertension and age on dCA are even less well documented. Limited studies have not demonstrated a consistent decline in autoregulation with age, although with conflicting results. Similarly, there is limited evidence of the effects of chronic hypertension on dCA and its relevance to development of cerebrovascular pathology, despite significant potential for new interventional targets.

### Physiologically guided treatment

Hypertension-related secondary prevention of cerebrovascular disease will be the focus of subsequent articles in this series, including intracerebral haemorrhage, recurrent ischaemic events and cognitive impairment. These intermediate pathophysiological targets between hypertension and clinical events provide new potential treatment targets. For example, calcium channel blockers and thiazide-like diuretics appear to reduce BP variability, but effects at different baseline blood pressure levels and by stroke aetiology are needed and there remains a need to define which antihypertensive medications are optimal in the secondary prevention of stroke.^113^

A number of trials are currently testing repurposed interventions to target specific aspects of these phenotypes. TREAT-SVDs is comparing the effects of amlodipine versus atenolol and losartan on cerebrovascular reactivity to inhaled carbon dioxide on MRI, and is expected to report in 2022/2023. OxHARP is testing phosphodiesterase inhibitors (sildenafil and cilostazol) on cerebral pulsatility and reactivity to carbon dioxide, using both transcranial ultrasound and MRI, with dCA as a secondary outcome. The completed but unreported PASTIS study (NCT02450253)assessed the effect of a single dose of tadalafil on white matter perfusion in the early phase after a lacunar stroke. LACI-1 tested implied improved cerebrovascular reactivity on MRI with either cilostazol or isosorbide mononitrate in lacunar stroke, leading to LACI 2 to test the same agents to prevent recurrent lacunar strokes and progression of white matter hyperintensities. This follows suggested benefits in larger clinical trials (PICASSO,^114^ CSPS^115^) with cilostazol reducing ischaemic stroke to an extent greater than expected from its antiplatelet effects, with no increased risk of haemorrhage. Finally, XILO-FIST has recently reported a null effect of allopurinol on progression of white matter hyperintensities, despite a small reduction in blood pressure.^116^ However, no trial has yet identified a reliable intervention that has lead on to definitive trials showing efficacy.

## Conclusion

The relationship between hypertension and stroke, and its reduction by appropriate blood pressure control, has significantly reduced its incidence. However, our developing understanding of the complex physiological effects of hypertension on the cerebral circulation, particularly in the context of cerebral SVD and its consequences, promises new treatment paradigms for the future.

## Non-standard Abbreviations and Acronyms

AF: Atrial fibrillation
ASL: Arterial spin labelling
ARI: Autoregulatory index
BBB: Blood brain barrier
BPV: Blood pressure variability
CAA: Cerebral amyloid angiopathy
CBF: Cerebral blood flow
CSF: Cerebrospinal fluid
CVR: Cerebrovascular reactivity
dCA: Dynamic cerebral autoregulation
MCA: Middle cerebral artery
MSNA: Muscle sympathetic nerve activity
NO: Nitric oxide
PVS: Perivascular spaces
PWV: Pulse wave velocity
RCT: Randomised controlled trial
RDN: Renal sympathetic denervation
SBP: Systolic blood pressure
SVD: Small vessel disease
TCD: Transcranial Doppler ultrasound
WMH: White matter hyperintensities
